# Late-Onset Hypokalemic Periodic Paralysis in an Adult Female With Type 2 Renal Tubular Acidosis: A Case Report

**DOI:** 10.7759/cureus.27695

**Published:** 2022-08-05

**Authors:** Vivian C Chukwuedozie, Tulika Garg, Hassan A Chaudhry, Saima H Shawl, Priya Mishra, Ngozi J Adaralegbe, Aadil Khan

**Affiliations:** 1 Internal Medicine, Ebonyi State University Medical School, Abakaliki, NGA; 2 Medicine, Government Medical College and Hospital, Chandigarh, IND; 3 Medicine, Medical University of Lublin, Lublin, POL; 4 Medicine, Chittagong Medical College Hospital, Chittagong, BGD; 5 Medicine, Ganesh Shankar Vidyarthi Memorial Medical College, Kanpur, IND; 6 Allied Health Sciences, University of Connecticut, Manchester, USA; 7 Department of Internal Medicine, Lala Lajpat Rai Hospital, Kanpur, IND

**Keywords:** type 2 renal tubular acidosis, autosomal recessive, acute flaccid muscle weakness, metabolic acidosis, hypokalemic periodic paralysis

## Abstract

Proximal renal tubular acidosis (type 2 RTA) is a metabolic disorder characterized by an inability of the proximal renal tubules to reabsorb bicarbonate, resulting in excessive urinary loss of bicarbonate. In return, this causes a standard anion gap metabolic acidosis with aberrant renal acidification, culminating in excessive urinary potassium loss and hyperchloremic metabolic acidosis. Several sources can induce potassium deficiency, ranging from slight abnormalities in potassium homeostasis to catastrophic and occasionally lethal circumstances. Hypokalemic periodic paralysis (HPP) manifests with broad muscle weakness and the absence of deep tendon reflexes, with the facial, bulbar, and respiratory muscles spared, and it subsequently requires the administration of intravenous potassium chloride to address the potassium imbalance. Some patients suffering from chronic potassium shortage may have periods of weakness. The clinical symptoms of distal RTA are identical to those of attacks induced by familial hypokalemic periodic paralysis (FPP). Muscle weakness may begin slowly and worsen over 24-48 hours to flaccid quadriplegia. RTA and FPP typically spare speech, swallowing, and ocular and respiratory muscles. As a result, families with RTA children must be aware of this risk. We present a case of HPP in a female caused by type 2 RTA.

## Introduction

Renal tubular acidosis (RTA) is a tubular disease associated with metabolic acidosis with an average anion gap. Several factors may lead to RTA, including inheritance, nephrotoxic drugs, and environmental factors. Proximal RTA is an autosomal recessive disease caused by mutations in sodium bicarbonate cotransporter located basolaterally in proximal renal tubules and urinary loss of glucose, phosphate ions, potassium ions, uric acid, and amino acids [1]. Type 2 RTA is an inability to reabsorb bicarbonate ions in the proximal tubules of the kidney, resulting in urinary loss of bicarbonate ions. Proximal RTA with Fanconi syndrome is caused by nephrotoxic drugs, including antiretrovirals, ifosfamide, and antiepileptic drugs [1]. Urinary loss of potassium ions in proximal RTA may cause hypokalemic paralysis, categorized into hypokalemic periodic paralysis (HPP) and hypokalemic nonperiodic paralysis [2]. Hypokalemic paralysis represents a set of conditions with low potassium and immediate flaccid muscle weakness. If the condition is not adequately corrected, problems linked to hypokalemia, such as hypokalemia-induced arrhythmias and respiratory paralysis, may lead to increased morbidity and fatality [3,4]. The most frequent cause in the Asian population includes thyrotoxic HPP, whereas, in Caucasians, the primary trigger constitutes familial periodic paralysis [5,6].

## Case presentation

A 37-year-old female without any comorbidities presented with a generalized weakness for the preceding four weeks. Her weakness worsened over the last four days. She had difficulty walking. She denied any history of trauma, fever, travel history, or alcohol use.

On physical examination, she had no pallor, icterus, cyanosis, clubbing, or lymphadenopathy. There were decreased bilateral deep tendon reflexes on systemic neurological examination, and the rest of the neurological examination was intact. There were no signs of meningeal irritation. The patient was able to pass urine, flatus, and stool. Her initial blood test results are shown in Table 1.

**Table 1:** Hematological parameters of the patient. MCH: mean corpuscular hemoglobin, MCHC: mean corpuscular hemoglobin concentration

Parameter	Laboratory value
Hemoglobin	12.9 (12-16.5) g/dL
MCH	25 (27-32) pg
MCHC	29.4 (31-35) g/dL
Red blood cell count	4.1 (4.2-5.4) million cells/mcL
White blood cell count	7,200 (4,000-11,000)/mcL
Platelet count	249,000 (150,000-450,000)/mcL

On admission, she was afebrile with a heart rate of 86 beats/minute, a respiratory rate of 16 breaths/minute, a blood pressure of 130/90, and oxygen saturation of 98% on room air.

To rule out possible etiologies of paralysis, some biochemical, pathological, and radiological tests were performed. Magnetic resonance imaging (MRI) of the lumbosacral spine was unremarkable. Serum calcium, serum magnesium, and thyroid function tests were also normal. Serum potassium was found to be low, and 24-hour urinary potassium was very high. Based on the investigations, a provisional diagnosis of hypokalemia and periodic paralysis was made. As on the ABG, blood pH was on the lower side, so we suspected type 2 RTA as one of the differentials. To confirm type 2 RTA, we did urine pH, which was >7.5, and fractional bicarbonate excretion, which was >15%, proving our provisional type 2 RTA. All the biochemical parameters are shown in Table 2.

**Table 2:** Biochemical parameters of the patient. LDH: lactate dehydrogenase, ALP: alkaline phosphatase, AST: aspartate aminotransferase, TSH: thyroid-stimulating hormone

Parameter	Laboratory value
Serum sodium	152 (135-147) mmol/L
Serum potassium	2.1 (3.5-5.1) mmol/L
Serum phosphorous	1.39 (1.12-1.45) mmol/L
Serum calcium	5.21 (4.5-5.6) mg/dL
Serum magnesium	1.8 (1.3-2.5) mmol/L
AST	39 (8-33) IU/L
ALP	812 (44-147) IU/L
LDH	345 (105-333) IU/L
Serum TSH	2.05 (7.35-7.45) IU/L

She was commenced on pantoprazole 40 mg, ondansetron 4 mg, normal saline, and ringer lactate. The patient was also given an oral solution for potassium with half a glass of water to treat low levels of potassium in the body. However, the patient did not improve on the oral solution, so intravenous (IV) potassium chloride (KCL) was started. After starting KCL, the patient's condition improved, and she was discharged.

## Discussion

Acute hypokalemic periodic paralysis (HPP) is characterized by widespread reversible muscular weakness and the absence of deep tendon reflexes, with the facial, bulbar, and respiratory muscles spared. Gender may play a role in the underlying causes of HPP [7]. Males are more likely to develop primary and thyrotoxic HPP. RTA is a sickness characterized by abnormal renal acidification, which results in potassium loss through the kidneys and metabolic acidosis. Poor proton secretion in distal nephrons results in distal RTA [8]. Abnormal bicarbonate reabsorption in proximal nephrons leads to proximal RTA. Muscle paralysis is uncommon in RTA, although hypokalemia is prevalent. The proximal kind of RTA has a much simpler HPP in comparison to the distal type, and the muscular weakness is subtle [9].

Excess loss of potassium from the kidney or gastrointestinal tract, or redistribution of potassium into cells, causes lower resting membrane potentials, further blocking the action potential [10]. This change could be due to increased sodium/potassium conductance ratio, sodium-potassium pump activity, or both [11]. Potassium deficiency manifests itself in various ways, from minor disruptions in potassium homeostasis to catastrophic and sometimes lethal situations [12]. Various factors can cause potassium deficiency, which can have a wide range of consequences on cellular metabolism [6]. Although familial hypokalemic periodic paralysis (FPP) is the most prevalent form of hypokalemic periodic muscle paralysis, certain patients with chronic potassium depletion may experience episodic weakness [8].

Hypokalemia can produce paralysis for a variety of reasons in a variety of disorders. Excessive excretion of potassium, or redistribution into cells, can cause hypokalemic paralysis [13]. Wasting of potassium through kidneys and accompanying paralysis has been observed in various conditions, including nephrotic syndrome, Fanconi syndrome, primary aldosteronism, and RTA [8]. Patients with hypokalemic muscular paralysis resulting from distal RTA have clinical characteristics that are indistinguishable from attacks caused by FPP [14]. In both conditions, muscle paralysis starts slowly and progresses over 24-48 hours to total flaccid quadriplegia [14]. Speech, swallowing, and ocular and respiratory muscles are frequently spared in RTA and FPP [14]. Muscle weakness in distal RTA might be misinterpreted, especially when the patient sees a physician who is not even a specialist in pediatric nephrology. Parents with RTA children should therefore be made aware of this possibility.

## Conclusions

Muscle paralysis has a broad spectrum of differentials and electrolyte abnormalities, particularly hypokalemia, which is one of the leading causes of periodic muscle paralysis. HPP with progressive symptoms should always be evaluated to identify an underlying etiology, and it is imperative to distinguish FPP from HPP. Our patient was diagnosed with HPP caused by type 2 RTA and responded well to potassium salts.
